# Evidence of progressive tissue loss in the core of chronic MS lesions: A longitudinal DTI study^☆^

**DOI:** 10.1016/j.nicl.2017.12.010

**Published:** 2017-12-08

**Authors:** Alexander Klistorner, Chenyu Wang, Con Yiannikas, John Parratt, Michael Dwyer, Joshua Barton, Stuart L. Graham, Yuyi You, Sidong Liu, Michael H. Barnett

**Affiliations:** a Save Sight Institute, Sydney Medical School, University of Sydney, Sydney, Australia; b Faculty of Medicine and Health Sciences, Macquarie University, Sydney, NSW, Australia; c Sydney Neuroimaging Analysis Centre, Sydney, NSW, Australia; d Brain and Mind Centre, University of Sydney, Sydney, NSW, Australia; e Royal North Shore Hospital, Sydney, NSW, Australia; f Buffalo Neuroimaging Analysis Center, University at Buffalo, Buffalo, NY, USA

**Keywords:** Multiple sclerosis, Lesions, Chronic demyelination, Diffusion

## Abstract

**Objective:**

Using diffusion tensor imaging (DTI), we examined chronic stable MS lesions, peri-lesional white matter (PLWM) and normal appearing white matter (NAWM) in patients with relapsing-remitting multiple sclerosis (RRMS) for evidence of progressive tissue destruction and evaluated whether diffusivity change is associated with conventional MRI parameters and clinical findings.

**Method:**

Pre- and post-gadolinium T1, T2 and DTI images were acquired from 55 consecutive RRMS patients at baseline and 42.3 ± 9.7 months later. Chronic stable T2 lesions of sufficient size were identified in 43 patients (total of 134 lesions). Diffusivity parameters such as axial diffusivity (AD), radial diffusivity (RD), mean diffusivity (MD) and fractional anisotropy (FA) were compared at baseline and follow-up. MRI was also performed in 20 normal subjects of similar age and gender.

**Results:**

Within the core of chronic MS lesions the diffusion of water molecules significantly increased over the follow-up period, while in NAWM all diffusivity indices remained stable. Since increase of AD and RD in lesional core was highly concordant, indicating isotropic nature of diffusivity change, and considering potential effect of crossing fibers on directionally-selective indices, only MD, a directionally-independent measure, was used for further analysis. The significant increase of MD in the lesion core during the follow-up period (1.29 ± 0.19 μm^2^/ms and 1.34 ± 0.20 μm^2^/ms at baseline and follow-up respectively, *P* < 0.0001) was independent of age or disease duration, total brain lesion volume or new lesion activity, lesion size or location and baseline tissue damage (T1 hypointensity). Change of MD in the lesion core, however, was associated with progressive brain atrophy (*r* = 0.47, *P* = 0.002). A significant gender difference was also observed: the MD change in male patients was almost twice that of female patients (0.030 ± 0.04 μm^2^/ms and 0.058 ± 0.03 μm^2^/ms in female and male respectively, *P* = 0.01). Sub-analysis of lesions with lesion-free surrounding revealed the largest MD increase in the lesion core, while MD progression gradually declined towards PLWM. MD in NAWM remained stable over the follow-up period.

**Conclusion:**

The significant increase of isotropic water diffusion in the core of chronic stable MS lesions likely reflects gradual, self-sustained tissue destruction in demyelinated white matter that is more aggressive in males.

## Highlights

• Chronic stable MS lesions were examined by using diffusion tensor imaging for evidence of progressive tissue destruction;
• Within the lesion core the diffusivity increased significantly over the follow-up period, while it remained stable in NAWM;
• Change of mean diffusivity in the lesion core was associated with progressive brain atrophy;
• This paper suggested progressive tissue destruction in demyelinated white matter.

## 1 Introduction

Multiple sclerosis (MS) is a chronic inflammatory neurodegenerative disease of the central nervous system (CNS). Axonal loss is now accepted as the major cause of irreversible neurological disability in MS. While acute inflammatory demyelination is thought to be a principal cause of axonal transection and subsequent axonal degeneration, other factors including axonal damage of permanently demyelinated axons and slow axonal burning at the lesion edge may also contribute to tissue damage, particularly in the progressive phase of the disease. However, contrary to the massive, rapid axonal damage that accompanies acute focal inflammation, chronic neurodegeneration is a continuous and slow process (Smith, 2006).

In the current study we used diffusion tensor imaging (DTI), a well-established and widely used technique for assessing white matter (WM) microstructure, to examine chronic stable MS lesions and peri-lesional white matter (PLWM) in patients with relapsing-remitting MS (RRMS) for evidence of progressive tissue alteration. Moreover, we evaluated whether diffusivity change is associated with conventional MRI parameters and clinical findings.

The limited number of published longitudinal DTI studies of chronic MS lesions are confounded by short duration or small sample size, often producing inconclusive or contradictory results (Caramia et al., 2002, Cassol et al., 2004, Garaci et al., 2007, Harrison et al., 2011a, Ontaneda et al., 2017, Rashid et al., 2008). Hence, in the current study we examined a sizable cohort of RRMS patients, who were followed-up for a relatively long observation period.

## 2 Material and methods

The study was approved by University of Sydney and Macquarie University Human Research Ethics Committees and followed the tenets of the Declaration of Helsinki. Written informed consent was obtained from all participants.

### 2.1 Subjects

Fifty-five consecutive patients with RRMS were enrolled. RRMS was defined according to revised McDonald criteria. Diffusion MRI scanning was performed twice – once at the time of enrolment and 42 months later (range 36–50 months). Diffusion MRI was also performed in 20 normal subjects of similar age and gender.

### 2.2 MRI protocol

The following sequences were acquired using a 3 T GE Discovery MR750 scanner (GE Medical Systems, Milwaukee, WI) as described previously in details: (Klistorner et al., 2016)

1. Pre- and post-contrast (gadolinium) Sagittal 3D T1 (voxel size 1 × 1 × 1 mm)
2. FLAIR CUBE (voxel size 1 × 1 × 1.2 mm);
3. Whole brain 64-directions diffusion weighted imaging with 2 mm isotropic acquisition matrix (voxel size 2 × 2 × 2 mm, b0 = 1000 s/mm^2^).

### 2.3 MRI image pre-processing

The baseline T1-weighted imaging was realigned to AC-PC orientation in MrVista package (Stanford University). Using FLIRT (FSL, FMRIB Software Library), follow-up T1 image was co-registered to baseline AC-PC space by applying transformation matrices derived from linear co-registration between baseline AC-PC aligned brain and follow-up native T1 brain images.

In parallel, diffusion MRI was corrected for motion and eddy-current distortion in FSL, then EPI susceptibility distortion was minimized by applying deformation maps generated from nonlinear co-registration between DWI b0 brain image and T1-weighted imaging at each time-point using ANTS (Advanced Normalization Tools).

Subsequently, tensor reconstruction was performed in MrDiffusion (MrVista, Stanford University). Baseline and follow-up tensor images were then linearly co-registered to corresponding T1 AC-PC images.

### 2.4 Lesion identification and analysis

Individual lesions were identified on the co-registered baseline T2 FLAIR images and semi-automatically segmented using JIM 7 software (Xinapse Systems, Essex, UK) by a trained analyst. The main criteria for lesion selection was a stable lesion appearance without visible signs of new acute inflammation (such as change in lesion shape) between baseline and follow-up as judged visually by two experienced observers.

**Fig. 1:**
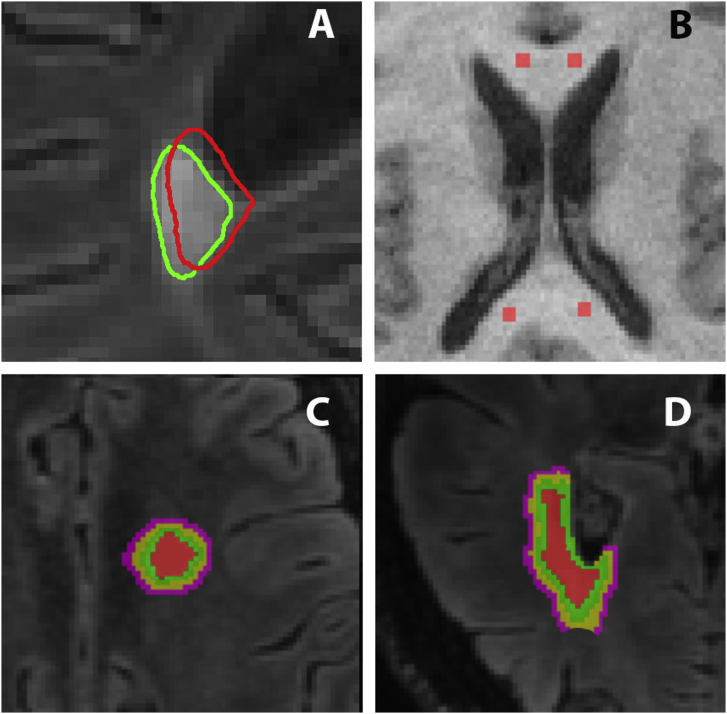
Lesion and NAWM ROIs. A. Examples of lesion position adjustment (lesion shifting outwards). Red contour: baseline lesion, green contour: follow-up lesion displayed on follow-up T2 FLAIR image. B. Example of 4 ROIs used for NAWM. C. Segmentation of lesional and perilesional white matter displayed on baseline T2 FLAIR image. Red colour represents lesion core, green colour represents lesion “rim” and PLWM shells are yellow and magenta. D. Example of removing CSF contamination from PLWM displayed on baseline T2 FLAIR image. (For interpretation of the references to colour in this figure legend, the reader is referred to the web version of this article.)

In order to compensate for the shift in position of follow-up lesions caused by ongoing brain atrophy the baseline lesion mask was projected onto the follow-up T2 FLAIR image and the position of every lesion in each slice examined and, where necessary, adjusted manually (Fig. 1A).

To minimize partial volume effects (Vos et al., 2011) and to isolate the potential effect of “slow burning” chronic inflammation and remyelination (Barkhof et al., 2003, Dal et al., 2017) which may occur at the lesion edge, the core of the lesion was identified by shrinking the segmented lesion mask in all directions by 1 voxel using the “eroding” function of JIM software. Only lesions with core appearing on > 2 consecutive slices and measuring larger than 65 mm^3^ (or 5 mm in diameter) were selected for analysis.

The eroded lesion mask was also applied to pre-contrast 3D T1-weighted images to quantify T1-hypointensity. In order to reduce inter-subject variability, the lesional hypointensity was normalised by the intensity of normal appearing WM (NAWM), which was measured using two additional 2 mm ROIs placed in both hemispheres in close proximity to the anterior horn of lateral ventricles. To perform normalization, the value of measured lesional hypointensity was multiplied by the ratio of averaged T1 hypointensity across all ROIs of all patients to average hypointensity of two ROIs of a particular patient.

In addition, to analyze diffusivity change around the lesion edge, a subset with lesion-free surrounding white matter was selected from the original lesion series. The inner-lesional area immediately surrounding the lesion core (lesion “rim”) and two expanding shells surrounding the lesion (peri-lesional white matter, PLWM) were generated. The thickness of each layer was 1 mm (Fig. 1C). PLWM ROIs were intersected with the white matter mask to avoid cerebrospinal fluid and grey matter contamination (Fig. 1D).

Furthermore, to analyze change of diffusivity in NAWM, four lesion-free ROIs (8 mm^3^ each) were created in the corpus callosum of each patient at least 1 cm away from any MS lesions (two in the genu and two in the splenium, Fig. 1B). A mean value across all four NAWM-ROIs was computed for the analysis at baseline and follow-up. Similar areas were selected in normal controls.

Axial, Radial and Mean Diffusivity (AD, RD and MD) and Fractional anisotropy (FA) were measured for each selected ROI at baseline and follow-up. Weighted-average diffusivity values of all ROIs of all selected lesions per patient (based on relative lesion volume) provided a single data point for each patient for plotting and statistical testing in patient-based analyses.

Gadolinum (Gd)-enhancing lesions at both time-points were excluded from the analysis.

### 2.5 Brain atrophy

Since resolution of pre-contrast T1 images was slightly altered during the course of the study, conventional techniques for the assessment of brain atrophy, based on T1 images, could not be used. Therefore, to estimate the rate of brain atrophy during the follow-up period we calculated lateral ventricular volume (LVV) using the recently proposed FLAIR-based NeuroSTREAM technique, which has been validated against conventional atrophy measures (Dwyer et al., 2017).

### 2.6 Statistical analysis

Statistical analysis was performed using SPSS 22.0 (SPSS, Chicago, IL, USA). Longitudinal changes were assessed using paired two-sample *t*-test. Comparisons between two groups were made using two-sample *t*-test. One-way ANOVA was used to assess differences between multiple groups. Pearson correlation coefficient was used to measure statistical dependence between two numerical variables. A Univariate General Linear Model was used to analyze potential effect of various factors on lesional diffusivity change. Progression rate was used as a dependent variable. *P* < 0.05 was considered statistically significant.

## 3 Results

Of 55 enrolled patients, 43 demonstrated stable individual T2 lesions with an eroded diameter > 5 mm or volume > 65 mm^3^ and were included in the analysis (Table 1). Data from single MRI scan of twenty normal control was also analysed.

**Table 1:** Patient demographics.

	Patients (*n* = 43)	Controls (*n* = 20)
Age (y)	42.1 ± 6.1	41.0 ± 9.1
Disease duration (y)	5.0 ± 3.0	n/a
Male/Female ratio	19:24	8:12
EDSS	1.38 ± 1.3	n/a
Follow-up duration (m)	42.3 ± 9.7	n/a

There were 134 individual lesions included in the final analysis. Each patient had between 2 and 4 lesions analysed that met the criteria. Forty-one patients (96%) were receiving disease-modifying therapy at the time of enrolment (3-beta-interferon 1b, 10-glatiramer acetate, 12-fingolimod, 7-natalizumab, 4-interferon-beta 1a, 3-dimethyl fumarate, 1-teriflunomide, 1-alemtuzumab).

Twenty-one patients developed new lesions during the follow-up period. While total brain lesion volume increased significantly (*P* = 0.0001), the volume of analysed lesions remained stable (*P* = 0.3). The longitudinal analysis is presented in Table 2. Within the core of chronic MS lesions the diffusion of water molecules significantly increased and FA decreased over the follow-up period, while in NAWM all diffusivity indices remained stable.

**Table 2:** Baseline and follow-up lesional data. AD, RD and MD measured as μm^2^/ms. *P* value reflects paired *t*-test.

	Baseline	Follow-up	*P* value
Total brain lesion volume	7313 ± 5862 mm^3^	8494 ± 6429 mm^3^	0.0001
Lateral ventricular volume	17.6 ± 8.5 ml^3^	18.9 ± 9.7 ml^3^	0.01
Average volume of analysed lesions	311 ± 371 mm^3^	310 ± 374 mm^3^	0.3
Lesional AD	1.62 ± 0.22	1.67 ± 0.23	< 0.0001
Lesional RD	1.13 ± 0.2	1.17 ± 0.21	< 0.0001
Lesional FA	0.234 ± 0.07	0.228 ± 0.07	< 0.0001
Lesional MD	1.29 ± 0.19	1.34 ± 0.20	< 0.0001
NAWM AD	1.59 ± 0.19	1.59 ± 0.17	0.6
NAWM RD	0.38 ± 0.09	0.38 ± 0.09	0.75
NAWM FA	0.71 ± 0.12	0.71 ± 0.11	0.48
NAWM MD	0.79 ± 0.03	0.79 ± 0.03	0.9

**Fig. 2:**
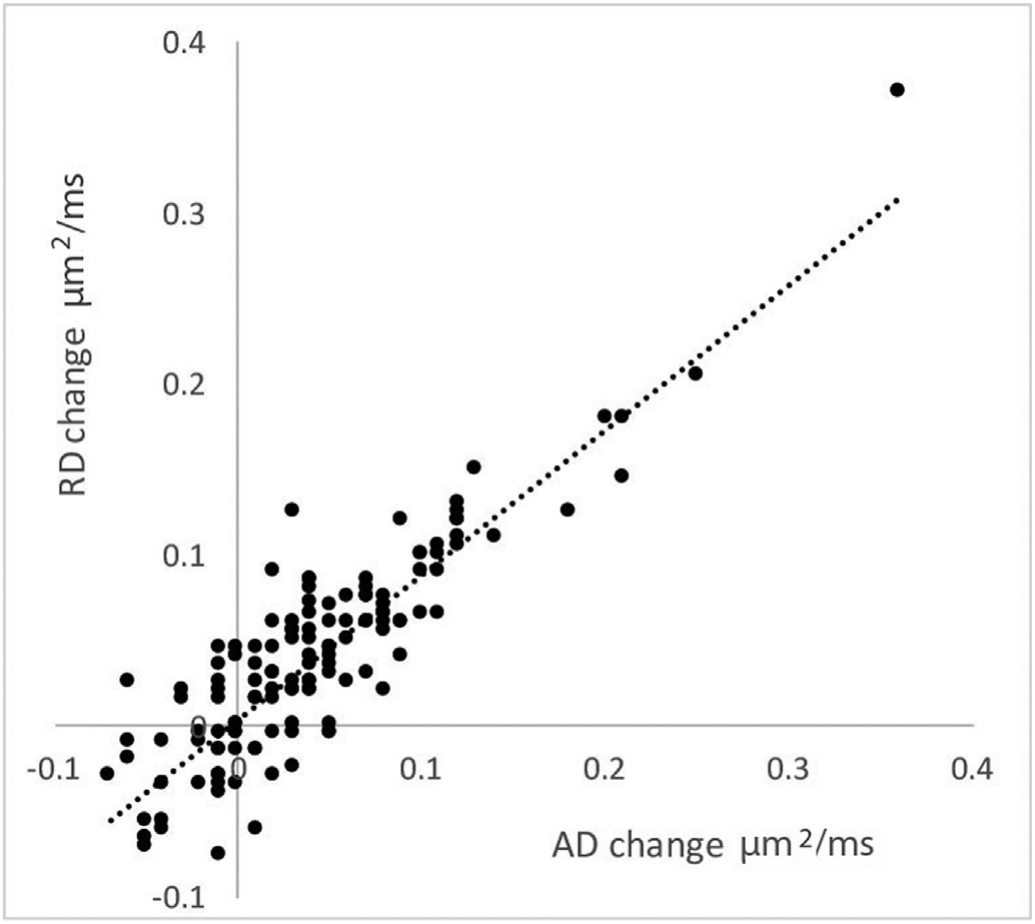
Correlation between AD and RD changes in individual lesions.

A highly significant correlation was observed between alteration in lesional AD and RD (*r* = 0.88, *P* < 0.001) (Fig. 2), indicating a concomitant rise of parallel and perpendicular diffusivity. These highly concordant changes in AD and RD support the concept that longitudinal alteration of diffusivity in the core of chronic MS lesions is largely isotropic. In addition, since a directionally-selective imbalance caused by various orientation of fibers within the voxel (Wheeler-Kingshott & Cercignani, 2009) and variation of diffusion anisotropy within the normal brain may significantly affect orientationally-sensitive indices (such as AD and RD) (Castriota-scanderbeg et al., 2003) only mean diffusivity (MD), a directionally-independent measure, was used for further analysis.

### 3.1 Patient-based analysis

a. Lesion core.

**Fig. 3:**
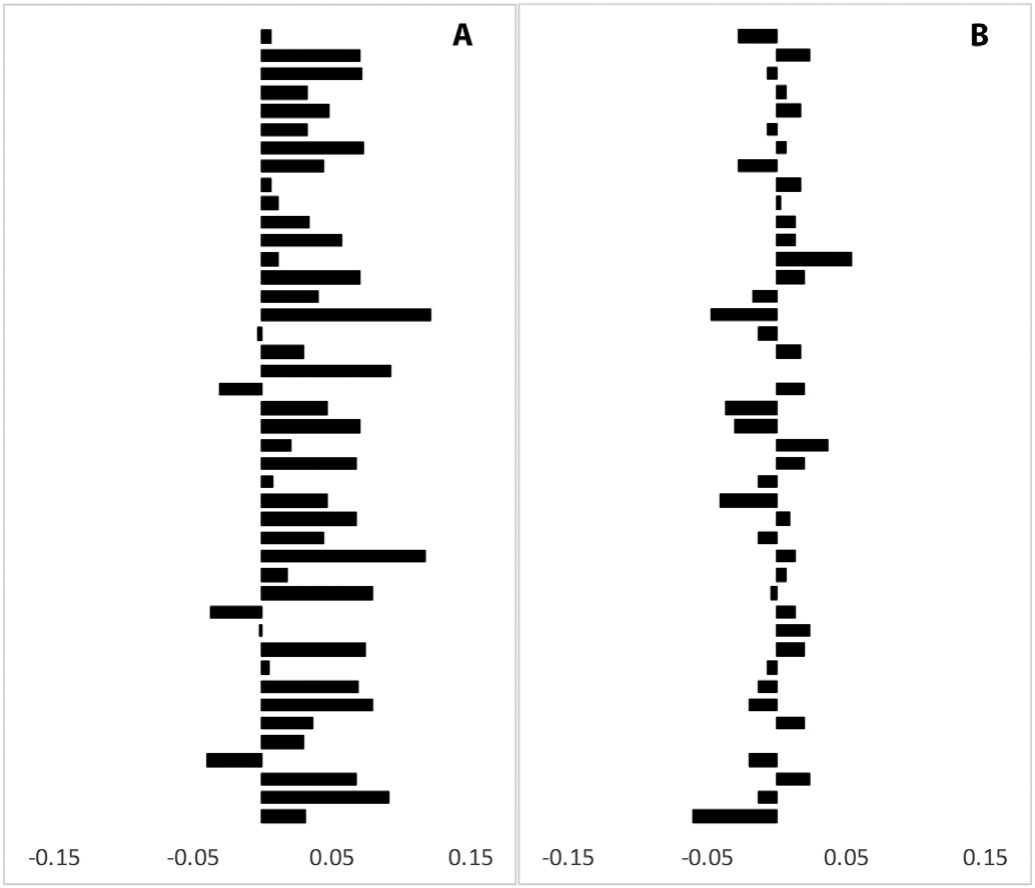
Change of MD in individual patients during follow-up period.

Overall there was significant increase (3.3%, *P* < 0.001, paired *t*-test) of averaged MD in the lesion core during follow-up, resulting in an annual change of 0.93%. No change was observed in NAWM (*P* = 0.8, paired *t*-test). Average MD change in lesion cores and NAWM for individual patients is presented in Fig. 3 (A, B).

Increase in MD was not associated with patient age, disease duration or duration of the follow-up (*r* = 0.02, 0.03 and 0.026, *P* = 0.9, 0.8 and 0.6 respectively, Pearson correlation), nor with total brain lesion volume at baseline or increase in brain lesion volume (*r* = 0.12 and 0.13, *P* = 0.2 and 0.5 respectively, Pearson correlation). In addition, grouping patients into those who developed new lesions during the follow-up period (21 patients) vs those who remained stable (22 patients) also did not show significant effect of new lesional activity on MD progression (0.38 vs 0.45 or 3.1% vs 3.5%, *P* = 0.6, *t*-test).

**Fig. 4:**
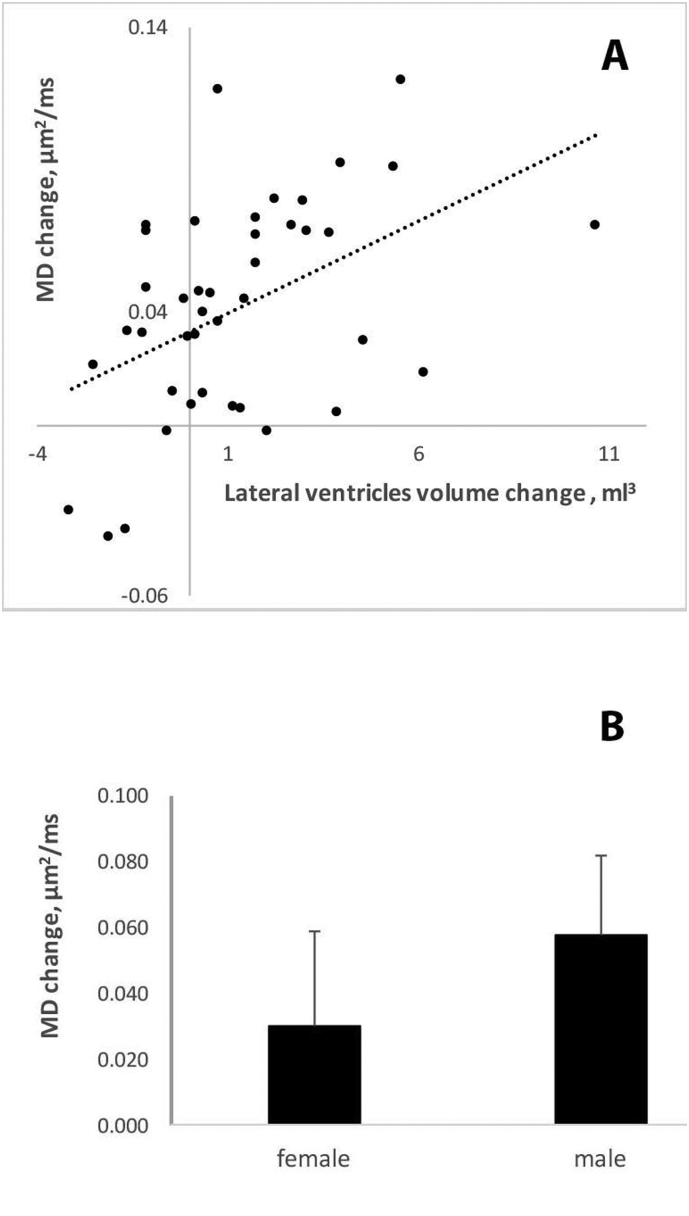
Effect of brain atrophy and gender on lesional diffusivity change: A. Association between increase of lateral ventricular volume and lesional MD change B. Change in lesional MD in male and female groups.

However, there was a significant correlation between increase in LVV and elevation of MD during the follow-up period (*r* = 0.47, *P* = 0.002) (Fig. 4A). We also observed a significant gender difference with regard to the level of MD change (*P* = 0.01, *t*-test). While in female patients average MD increased by 0.030 ± 0.04 μm^2^/ms or 2.3%, the diffusivity change was almost twice as large in male patients (0.058 ± 0.03 μm^2^/ms or 4.5%) (Fig. 4B).

Since the rate of brain atrophy is typically faster in patients with high T2 lesion load or patients who develop new lesions (Radue et al., 2015), the associations were examined between baseline lesion volume, increased lesion load during study follow-up and alteration of brain atrophy in the study cohort. Both lesional measures correlated significantly with change in LVV (*r* = 0.35, *P* = 0.03 and *r* = 0.42, *P* = 0.007 respectively). In addition, we also observed significant correlation between baseline EDSS and increase of MD in the lesion core (*r* = 0.33. *P* = 0.036). Therefore, Univariate General Linear analysis was performed adjusting for those parameters. Association of MD lesion change with both gender and brain atrophy survived multiple comparisons of the model after age, disease duration, duration of the follow-up, baseline EDSS, baseline lesion volume and lesional activity (i.e. brain lesion volume increase) during the follow-up period were added to the model (*P* = 0.038 and 0.019, F = 5.4 and 7.7 respectively). No other factors showed significance.

b. Sub-analysis of peri-lesional white matter (PLWM).

**Fig. 5:**
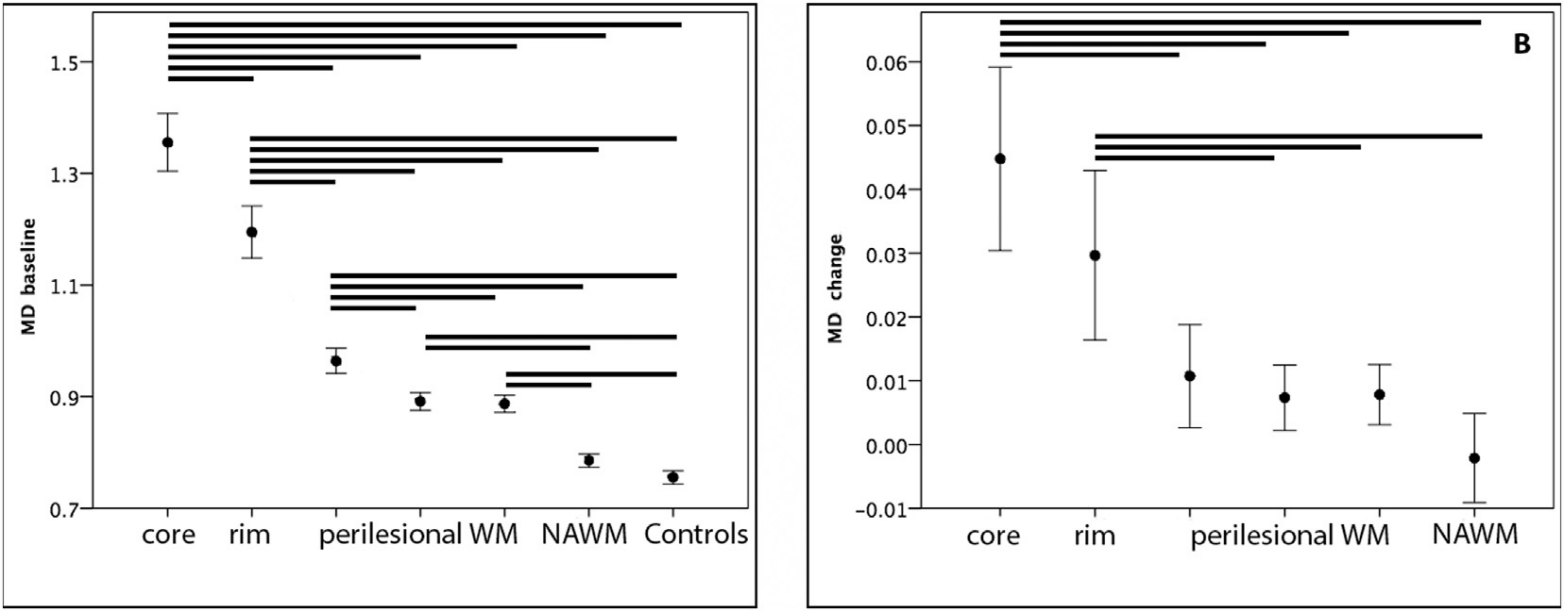
Sub-analysis of lesional and peri-lesional white matter. A. Baseline MD in lesion core, lesion rim, PLWM, NAWM and normal controls (vertical scale-μm^2^/ms, error bar ± 2SE). B. MD progression in lesion core, lesion rim, PLWM and NAWM. (vertical scale-μm^2^/ms, error bar ± 2SE). Horizontal bars represent statistically significant difference (*P* < 0.05).

There were 101 individual lesions from 38 patients selected for sub-analysis with PLWM meeting the lesion-free criteria. As expected, baseline data demonstrated highest MD value in the lesion core with gradual tapering towards PLWM. Post-hoc analysis showed a significant difference between all lesional and peri-lesional ROIs (*P* < 0.001 for all, except between two PLWM shells (*P* = 0.02), one-way ANOVA). In addition, MD in NAWM was significantly lower compared to all lesional and perilesional ROIs (*P* < 0.001 for all, one-way ANOVA) (Table 3, Fig. 5A).

**Table 3:** Peri-lesional white matter MD analysis. MD measured as μm^2^/ms.

	Baseline	Follow-up	*P* value
Lesional core MD	1.35 ± 0.16	1.40 ± 0.16	< 0.001
Lesional rim MD	1.20 ± 0.14	1.23 ± 0.15	< 0.001
PLWM MD:			
Inner shell	0.96 ± 0.07	0.97 ± 0.07	0.01
Outer shell	0.89 ± 0.05	0.90 ± 0.05	0.01
NAWM MD	0.79 ± 0.04	0.79 ± 0.04	0.7
Controls MD	0.76 ± 0.03	n/a	

While the baseline MD of NAWM in MS patients was higher compared to similar regions in normal controls (0.79 ± 0.04 vs 0.76 ± 0.03, *P* = 0.001, Student *t*-test), this difference did not survive multiple comparisons of ANOVA.

Longitudinal analysis (Table 2, Fig. 5B) revealed significant progression of MD in all lesional and PLWM ROIs. The largest increase was observed in the lesion core (0.0448 ± 0.04 μm^2^/ms, *P* < 0.001, paired *t*-test), while change in the lesion rim was less (0.0297 ± 0.04 μm^2^/ms, P < 0.001, paired *t*-test). PLWM ROIs demonstrated small, but still significant MD change (0.0107 ± 0.03 μm^2^/ms and 0.0073 ± 0.02 μm^2^/ms for inner and outer shells respectively, *P* = 0.01 for both, paired *t*-test). In contrast, MD in NAWM ROI remained stable over the follow-up period (*P* = 0.7, paired *t*-test).

One-way ANOVA comparison of MD incremental change between all ROIs demonstrated significantly larger MD increase in the lesion core and rim area compared with NAWM (*P* < 0.001 for both, Tukey post-hoc test) and PLWM ROIs (except for the difference between lesion rim and innermost shell) (*P* < 0.001 and *P* < 0.01 for lesion core and rim respectively, Tukey post-hoc test). The difference between MD change in PLWM and NAWM, however, did not reach statistical significance (*P* = 0.4 and 0.7 for inner and outer shells respectively, Tukey post-hoc test) (Fig. 5B).

### 3.2 Analysis of individual lesions

Analysis of individual lesions demonstrated no association between MD change and lesion size (*r* = 0.045, *P* = 0.6). Since lesions abutting ventricles are potentially susceptible to partial volume effects (particularly in cases of progressive ventricular enlargement) we compared MD change in periventricular lesions (*n* = 66) and lesions completely surrounded by NAWM (*n* = 68). No statistical difference (0.42 vs 0.43, *P* = 0.9) was demonstrable.

Baseline lesional T1-hypointensity and MD values in individual lesions varied widely (15,401 ± 2711 units and 1.29 ± 0.19 μm^2^/ms respectively), though these two parameters correlated significantly between each other (*r* = 0.63, *P* < 0.001), suggesting an extensive spectrum of tissue damage. However, no correlation was observed between these baseline metrics and progressive MD change during the follow-up period (*r* = 0.1, *P* = 0.27 and *r* = 0.03, *P* = 0.23 for T1-hypointensity and MD respectively).

## 4 Discussion

The current study addresses longitudinal microstructural changes within chronic stable demyelinated MS lesions using DTI.

While novel diffusion-based methods are currently emerging (e.g. NODDI, DBSI), they are difficult to implement in a setting of large longitudinal study and their clinical value is yet to be proven. Conventional DTI technique, on the other hand, can easily be imbedded into clinical MRI protocol and implemented on a standard scanner. DTI modelling, however, relies heavily on the assumption of voxel-based fiber coherency. Hence, due to extensive fiber crossing (Jeurissen et al., 2013), this model does not adequately estimate orientationally-selective diffusivity measures such as AD and RD outside of highly coherent fiber tracts(Taipel et al., 2014, Wheeler-Kingshott and Cercignani, 2009). MD, on the other hand, represents a non-directional measure of diffusivity and, therefore, may be more appropriate for voxel-based assessment of white matter microstructure when the complexity of underling fibers is unclear. This is particularly relevant in a case of highly parallel alteration of AD and RD in the lesion core observed in the current study. The use of MD as a marker of structural change in white matter is further supported by the close association found between level of MD and lesional T1 hypointensity, an imaging metric that is closely associated with the degree of tissue destruction within MS lesions(van Waesberghe et al., 1999, van Walderveen et al., 1998). This is consistent with previous DTI studies, which demonstrated highest diffusivity in lesions with severe tissue loss (Scanderbeg et al., 2000, Filippi et al., 2000, Droogan et al., 1999, Bammer et al., 2000).

The main finding of the current study was a significant increase of MD within and in a close proximity of chronic stable brain lesions in patients with RRMS over the 3–4 years period of observation. The diffusion increment was largest in the lesional core with gradual reduction towards the PLWM. While water diffusion in NAWM was marginally higher at baseline compared to similar regions in normal controls, it remained stable during follow-up period, which is consistent with previous reports (Agosta et al., 2007, Ontaneda et al., 2017, Rashid et al., 2008).

The observed increase of water diffusion in the lesional core was associated with progressive brain atrophy and demonstrated a male gender preponderance. It was largely isotropic and independent of:

– the degree of pre-existing tissue damage within the lesion (as determined by the absence of correlation with the degree of T1 hypo-intensity and the level of MD at baseline);
– the degree of inflammatory disease activity during the follow-up period (since there was no association with the incidence of new lesions or increase in total brain lesion volume);
– Wallerian degeneration (since no diffusivity increase was observed in NAWM);
– patient age and disease duration;
– “slow burning” chronic inflammation and potential remyelination (since both are more common at the lesion rim, which demonstrated considerably smaller diffusivity change);
– partial volume effect (since there was no association with lesion location);
– lesion size.

Studies related to longitudinal diffusivity change in MS are scarce, tend to be of short duration, small sample size and typically do not show significant progression of diffusivity indices in both NAWM and whole brain (including MS lesions) (Cassol et al., 2004, Garaci et al., 2007, Rashid et al., 2008). While two recently published longitudinal DTI studies of comparable length have demonstrated some diffusivity alteration in MS patients over time, the nature of the observed change was contradictory (Caramia et al., 2002, Miller et al., 2014).

Harrison et al. (2011b) reported that an increase in water diffusion is driven exclusively by change in RD. Conversely, the study published by Ontaneda et al. (2017) demonstrated that progression is solely dependent on increase in AD. These findings are contrary to the concomitant increase of lesional AD and RD in our study. Methodological differences may account for the observed discrepancies. Thus, Harrison et al. (2011a) studied patients with all MS subtypes and both NAWM and lesions were co-analysed with no correction for brain atrophy or the appearance of new lesions. In addition, follow-up of some patients was as short as 6 months. Small sample size, gender composition, treatment regime and, in particular, change in the DTI protocol during follow-up period may have influenced the results reported by Ontaneda et al. (2017). Isolation of the lesion core, as opposed to the analysis of the entire lesion (Klistorner et al., 2016) may have also contributed to a more significant diffusivity change (and potentially better sensitivity) observed in our study by minimising an impact of “slow burning” chronic inflammation and remyelination (Barkhof et al., 2003, Dal et al., 2017). In addition, a thorough adjustment of lesion position at follow-up to compensate for the development of brain atrophy helped to eliminate contamination by partial volume effect caused by expanding ventricles. Accelerated brain atrophy in patients with MS is a significant confounding factor in longitudinal studies of brain imaging (Bermel and Bakshi, 2006) and its elimination may have a critical effect on the quality of the longitudinal lesion analysis.

While alteration of MD in chronic MS lesions is dependent on the level of extra-cellular space expansion and degree of demyelination (Sbardella et al., 2013), the latter is likely to remain stable, since axons in the core of chronic non-active MS lesions are believed to be fully demyelinated and partial remyelination, when present, is typically limited to the lesional rim and begins in the immediate aftermath of acute inflammatory demyelination) (Barkhof et al., 2003, Klistorner et al., 2016). New bouts of active inflammatory demyelination in the core of chronic lesions are also unlikely since firstly, axons are already demyelinated; secondly, the observed diffusivity increase was predominantly isotropic (contrary to the preferential increase of RD expected from demyelination); and finally, no relationship was found between progressive MD change and brain lesional activity during follow-up period.

Hence, we argue that the isotropic nature of progressive diffusivity increase in the core of chronic MS lesions most likely reflects further enlargement of the extra-cellular space caused by ongoing tissue loss. In chronic lesions this tissue is largely represented by demyelinated axons and glia. Therefore, providing that glial elements remain unaltered, the tissue loss is likely to reflect an axonal demise.

Of relevance is that several lines of evidence suggest permanent demyelination may contribute to accelerated axonal degeneration by rendering axons vulnerable to physiological stress (Bruck, 2005, Kornek et al., 2000). Chronic demyelination increases the energy demand of axonal conduction, ultimately compromising axoplasmic ATP production, leading to an ionic imbalance and Ca^2 +^-mediated axonal degeneration (see (Correale et al., 2017) for review). In addition, lack of trophic support from myelin or oligodendrocytes and disruption of normal axon-myelin interactions may also lead to degeneration of chronically demyelinated axons (Peterson et al., 2005, Trapp et al., 1999). Loss of demyelinated axons may also be exacerbated by activation and proliferation of astrocytes (Correale et al., 2015, Liddelow et al., 2017).

An association between the rate of ventricular enlargement and progressive increase of the diffusivity in the lesion core supports the notion that neurodegenerative processes within chronic demyelinated lesions may contribute to atrophy of brain tissue - presumably through the mechanism of Wallerian degeneration. While the persistent clinical features of early RRMS are primarily determined by massive axonal transection within focal inflammatory lesions, this neurodegenerative component of the disease is progressively ‘unmasked’ with increasing disease duration and exhaustion of neuro-axonal reserve (Bermel and Bakshi, 2006).

We also observed a more prominent increase in lesional MD in males versus females over the course of the study. This gender disparity may reflect a difference between males and females in relation to disease evolution. While the incidence of MS is greater in females (Compston & Coles, 2008), males in general have a more severe disease phenotype with worse clinical outcomes and faster accumulation of disability (Ribbons et al., 2015, Shirani et al., 2012, Tomassini and Pozzilli, 2009). The pathophysiological basis of this gender disparity may be related to a differential effect of sex hormones on neuroprotection and de/remyelination: female sex hormones may confer a higher susceptibility to autoimmunity (see (Harbo et al., 2013, Miller et al., 2014) for review), whereas male hormones appear to protect against the development of autoimmunity (Eikelenboom et al., 2009).

The small, but significant increase of MD in PLWM during the follow-up period, may represent the combined result of Wallerian degeneration caused by axonal transection in the lesional core and progressive changes in small lesions which are beyond the resolution of the current MRI technique (Filippi et al., 2000). Alternatively, it may reflect ongoing low-grade inflammation at the edge of chronic slow-expanding lesions (Dal et al., 2017, Prineas et al., 2001, Reynolds et al., 2014). This inflammation is characterized by a rim of activated microglia and axonal transection and can spread as far as several millimeters from the lesion edge (Prineas et al., 2001, Frischer et al., 2016).

This study has several limitations. Specifically, the real age of individual lesions remains unknown. This can make it difficult to estimate the true significance of the relationship between severity of tissue damage at baseline and longitudinal diffusivity change. Another limitation is the lack of longitudinal DTI data in control subjects. However, several studies have demonstrated minimal, if any, time-related change of DTI indices in normal subjects of comparable age (Rashid et al., 2008, Tur et al., 2016). In addition, stable diffusivity in NAWM served as an “internal” control reassuring negligible, if any age-related change of diffusivity and reliability of the DTI protocol during the study. Finally, the relatively small sample size precluded assessment of the potential impact of disease modifying therapies on the results of the study.

The findings of the study have several important implications. Firstly, progressive tissue damage occurring in the core of chronic inactive MS lesions is associated with progressive brain atrophy and likely reflects an accelerated loss of demyelinated axons. This highlights the imperative to develop pro-remyelinating therapies. Secondly, gender differences must be taken into account in clinical trials assessing the potential neuroprotective or pro-remyelinating effects of particular therapy. Thirdly, controlling for the morphological effects of brain atrophy is critically important for accurate longitudinal analysis of MS lesions.

## Author contributions

1) Conception and design of the study (AK, CY, SG, MB); 2) acquisition and analysis of data (AK, CW, CY, JP, MD, JB, SL, YY, MB, SL); 3) drafting a significant portion of the manuscript or figures (AK, CW, SG, YY, MB).

## Conflicts of interests

Nothing to report.
